# The Effect of Glucose or Fructose Added to a Semi-solid Meal on Gastric Emptying Rate, Appetite, and Blood Biochemistry

**DOI:** 10.3389/fnut.2018.00094

**Published:** 2018-10-10

**Authors:** Gethin H. Evans, John McLaughlin, Adora M. W. Yau

**Affiliations:** ^1^School of Healthcare Science, Manchester Metropolitan University, Manchester, United Kingdom; ^2^Division of Diabetes, Endocrinology and Gastroenterology, School of Medical Sciences, University of Manchester and Salford Royal Hospitals, Manchester, United Kingdom

**Keywords:** glucose, fructose, appetite, gastric emptying rate, blood glucose, triglycerides

## Abstract

The ingestion of fructose is of interest due to previously reported differences in gastrointestinal, appetite, and metabolic effects when compared to glucose ingestion when ingested in liquid solution. The aim of this study was to examine these variables when fructose and glucose are added to a semi-solid meal. Seven healthy male participants completed three experimental trials involving the ingestion of 300 mL of semi-skimmed milk mixed with 40 g of instant porridge mix (CON) and with the addition of either 40 g of glucose (GLU) or fructose (FRU). Subjective feelings of appetite were assessed for 2 h after ingestion with blood samples collected at regular intervals. Gastric emptying rate was assessed using the ^13^C breath test method. Half emptying time was not different between trials (CON = 159 ± 51 min; GLU = 197 ± 46 min; FRU = 198 ± 67 min: *P* = 0.117). No differences were observed for any subjective measurements of appetite (*P* > 0.05) while blood glucose was elevated (*P* < 0.05) 20 min after ingestion on both GLU and FRU with this tending to be higher on GLU than FRU. FRU resulted in greater (*P* < 0.05) blood lactate concentrations than on the other trials. The results of this study demonstrate that gastric emptying rate of glucose and fructose is similar when ingested in a semi-solid meal. In addition, there is little difference in appetite response between these sugars, however, there are some differences in metabolic response which deserve further study.

## Introduction

The role of simple sugar ingestion in the etiology of chronic disease states has received a lot of attention in recent times. Of particular interest has been the potential negative effects of dietary fructose, given that the ingestion of this monosaccharide has increased rapidly (1), and it has been suggested that this may play a role in the development of obesity and metabolic syndrome (1, 2). Fructose is found in a number of organic food products, including fruit. However, it has increasingly been added to products due to its sweet taste primarily in the form of sucrose or, particularly in the United States, as high fructose corn syrup.

A number of key differences have been observed regarding the gastrointestinal, metabolic, and appetite stimulating effects of fructose in comparison with glucose. The rate of gastric emptying of an ingested substance is an important consideration as this is one determinant of the rate an ingested sugar is available for absorption in the small intestine and appearance in the circulation. In addition, gastric distension is an important consideration in the processes of satiation and satiety (3). Previous research (4) has demonstrated that, when ingested in a liquid form, gastric emptying rate is linearly related to energy density. Similarly, other research (5) has demonstrated that increasing the quantity of glucose in a solution reduces gastric emptying rate. Comparatively little is known, however, about the gastric emptying rate of fructose. Horowitz et al. (6) observed that gastric emptying rate of fructose was faster than glucose, prior to a period of glucose supplementation, when ingested in liquid form. These results are similar to those obtained by Yau et al. (7, 8). These results yield interesting observations when monosaccharides are ingested in liquid form. However, no information is currently available on whether ingesting monosaccharides in a semi-solid or solid meal results in differences in gastric emptying rate. Solid meals are emptied from the stomach at a slower rate than liquids (9) and, in particular, a longer lag phase in order to allow tituration of ingested substances to occur. Related to this, the proximal stomach appears to have a greater role in controlling gastric emptying rate of solids while the distal stomach has a greater role in controlling gastric emptying rate of liquids (10). It is, therefore, of interest to study the effects of adding sugar to liquids, semi-solids and solids on gastric emptying rate as effects may differ.

Previous research has suggested that the ingestion of fructose results in different metabolic responses compared to when glucose is ingested. Specifically, Kong et al. (11) observed that the ingestion of 75 g of fructose resulted in an increase in blood glucose, glucagon-like-peptide 1, and insulin concentrations but all to a lesser extent than when an equivalent amount of glucose was ingested. Similarly, Chong et al. (12) reported that ingestion of 0.75 g/kg body mass of fructose resulted in a greater increase in circulating triglyceride concentration than an equivalent quantity of glucose, while Teff et al. (13, 14) reported that when dietary fructose was increased during a 24 h period circulating concentration of leptin was reduced. Similarly, Luo et al. (15) reported that ingestion of 75 g of fructose activated certain brain centers that were related to food choice and preference than an equivalent amount of glucose. Yau et al. (16) reported that ingestion of smaller quantities of fructose (36 g) significantly increased blood lactate and serum glucose dependent insulinotropic peptide (GIP) concentrations but had little impact on other metabolic markers or appetite when ingested in liquid form. These observations, in combination with those of chronic feeding studies (17, 18), provide some potential mechanisms for the reported relationships between excess fructose intake and risk of obesity and metabolic diseases. An important consideration, however, is that in these studies monosaccharides were delivered in liquid form and observations could be different if a monosaccharide is added to a semi-solid or a solid meal given that gastric emptying of solid and semi-solid foods is slower than for liquids. There is a clear effect of gastric emptying rate on glycaemic response of food ingestion (19) with about 35% of variability in glycaemic response accounted for by gastric emptying rate (20). Given the difference in gastric emptying rates observed between solids and liquids (9), it is important to determine the effect that adding sugar to liquids, semi-solids and solids have on metabolic parameters.

The aim of this study was to investigate whether the addition of glucose or fructose to a semi-solid meal influences gastric emptying rate, subjective feelings of appetite, and metabolic blood parameters.

## Methods

Seven healthy male participants (Mean ± SD: Age = 27 ± 5 yr; Height = 179 ± 6 cm; Body Mass = 85.3 ± 11.0 kg; Body Fat = 23.0 ± 8.2%) volunteered to participate in this investigation. Participants completed a medical screening questionnaire prior to experimental trials to ensure they were free from any known history of conditions or disease before providing written informed consent. This study was approved by the Institutional Ethical Advisory Committee.

### Experimental procedure

Each participant completed three experimental procedures that were completed in a single blind randomized order following pre-trial standardization and were separated by a period of at least 7 days. Participants recorded their diet and physical activity in the 24 h preceding the first experimental trial and they were asked to replicate these activities in the 24 h before the subsequent experimental trials. Adherence to this protocol was provided verbally to the researchers upon arrival at the laboratory. Experimental trials began between 0800 and 1000 in the morning following an overnight fast from 2100 with the exception of ingestion of 500 mL of water ~1 h before arrival at the laboratory in an attempt to standardize pre-trial hydration status.

Following arrival at the laboratory, participants completely voided their bladder before an intravenous cannula was inserted into an antecubital vein. This was used for all blood sample collections and was kept patent by the infusion of isotonic saline following each draw. Following collection of a blood sample, a visual analog scale (VAS) was used to assess participants subjective feelings of hunger, fullness, and prospective food consumption. This involved participants indicating on a 10 cm scale (with 0 cm representing “not at all” and 10 cm representing “very”) their subjective feeling at that time. Participants then ingested a semi-solid meal consisting of 300 g of heated semi-skimmed milk mixed with 40 g of commercially available instant porridge (Ready Brek, The Weetabix Food Company, Kettering, United Kingdom) mix (CON) or the same meal with 40 g of added glucose (GLU) or 40 g of added fructose (FRU). The composition of the test meal is detailed in Table 1. Participants were instructed to ingest the meal evenly over a 15 min period. Following ingestion, VAS questionnaires were completed at 10 min intervals and blood samples were collected at 20, 40, 60, 90, and 120 min after ingestion. Following the final data collection point, the cannula was removed and participants were free to leave the laboratory.

**Table 1:** Energy (kcal), carbohydrate (g), fat (g), protein (g), and added sugar (g) of the control (CON), glucose (GLU), and fructose (FRU) test meals.

	**CON**	**GLU**	**FRU**
Total energy (kcal)	298	458	458
Total carbohydrate (g)	37.4	77.4	77.4
Total fat (g)	8.9	8.9	8.9
Total protein (g)	15.6	15.6	15.6
Added sugar (g)	0	40	40

### Gastric emptying measurement and sample analysis

Gastric emptying rate was assessed using the ^13^C breath test method. 100 mg of ^13^C sodium acetate (Cambridge Isotope Laboratories Inc., Andover, USA) was added to the test meal and end expiratory volume breath samples were collected before ingestion and at 10 min intervals after ingestion for 120 min. Breath samples were analyzed using non-dispersive infrared spectroscopy (IRIS, Wagner, Germany) for ^13^CO_2_:^12^CO_2_ ratio with the difference in this ratio from baseline levels being presented as Delta Over Baseline (DOB) values. Using onboard software, the time taken to empty half of the test meal (T_1/2_) and the time of maximal emptying rate (T_lag_) were calculated.

Blood samples were collected into serum collection vacutainers and centrifuged at 1500 x g for 15 min at 4°C before serum was separated and stored at −80°C until batch analysis was performed at the end of the data collection period. Serum samples were analyzed for blood glucose, lactate and triglyceride concentrations using a clinical chemistry analyser (Randox Daytona, Crumlin, UK). All analysis was performed in duplicate.

### Statistical analysis

Incremental area under the curve (iAUC) for blood biochemistry markers were calculated using the trapezoid method. Differences in T_1/2_, T_lag_, and iAUC were assessed using one factor repeated measures ANOVA with Bonferroni adjusted paired *t*-tests as *post-hoc* analysis when appropriate. Two factor repeated measures ANOVA was used to assess main effects of trial, time and interaction for DOB values, appetite ratings, and blood biochemistry markers. *Post-hoc* analysis, where appropriate, consisted of one factor repeated measures ANOVA and paired *t*-tests with Bonferroni adjustments. The Greenhouse-Geisser epsilon was used to correct for violations of sphericity. Degree of Freedom (d.f.) and *F*-values for ANOVAs are presented. A critical value of 0.05 was used and all analysis was completed using IBM SPSS version 23. Data is presented as Mean ± SD.

## Results

### Gastric emptying rate

T_1/2_ (*P* = 0.117) and T_lag_ (*P* = 0.087) data are presented in Table 2. Two factor ANOVA on DOB data demonstrated no main effect of trial (d.f. = 1.165; *F* = 0.006; *P* = 0.958), a main effect of time (d.f. = 1.450; *F* = 42.209; *P* < 0.001), and no interaction effect (d.f. = 1.910; *F* = 0.130; *P* = 0.871).

**Table 2:** T_1/2_ and T_lag_ (minutes) for the control (CON), glucose (GLU), and fructose (FRU) test meals.

	**CON**	**GLU**	**FRU**	***P*-value**
T_1/2_	159 ± 51	197 ± 46	198 ± 67	0.117
T_lag_	78 ± 21	88 ± 26	89 ± 23	0.087

### Subjective feelings

**Figure 1:**
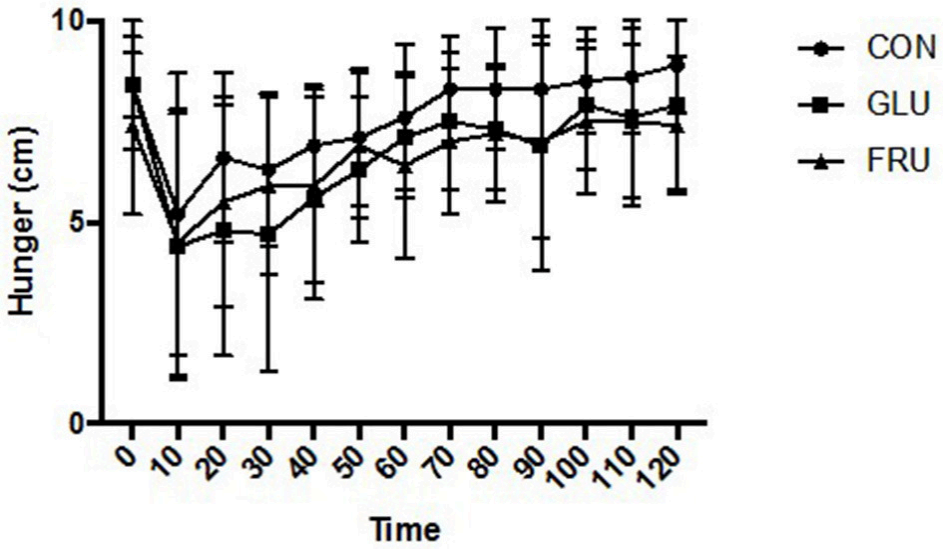
Subjective feelings of hunger during CON, GLU and FRU trials. 0 cm refers to “I am not hungry at all” and 10 cm refers to “I have never been more hungry.” Data are Mean ± SD.

**Figure 2:**
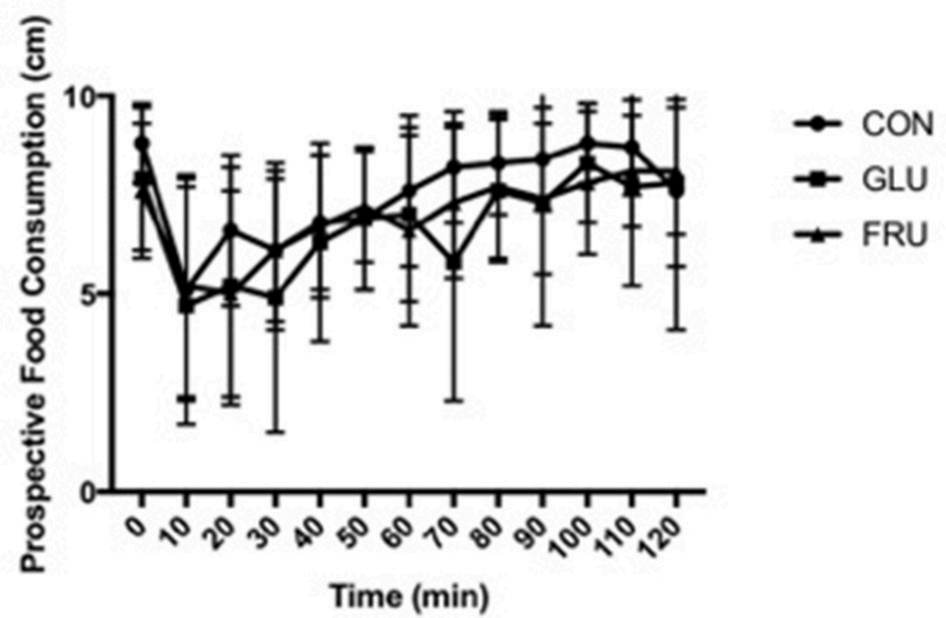
Prospective food consumption during CON, GLU and FRU trials. 0 cm refers to “Nothing at all” and 10 cm refers to “A lot.” Data are Mean ± SD.

Two factor ANOVA demonstrated no main effect of trial (d.f. = 1.445; *F* = 2.627; *P* = 0.136), a main effect of time (d.f. = 2.491; *F* = 10.403; *P* = 0.001), and no interaction effect (d.f. = 2.744; *F* = 1.057; *P* = 0.389) for subjective feelings of hunger (Figure 1). No main effect of trial (d.f. = 1.214; *F* = 0.775; *P* = 0.432), a main effect of time (d.f. = 3.849; *F* = 8.502; *P* < 0.001), and no interaction effect (d.f. = 2.280; *F* = 1.051; *P* = 0.385) was observed for subjective feeling of fullness. Similarly, no main effect of trial (d.f. = 1.203; *F* = 1.255; *P* = 0.311), a main effect of time (d.f. = 3.200; *F* = 11.636; *P* < 0.001), and no interaction effect (d.f. = 3.565; *F* = 0.909; *P* = 0.467) was observed for prospective food consumption (Figure 2).

### Serum glucose, lactate, and triglycerides

**Figure 3:**
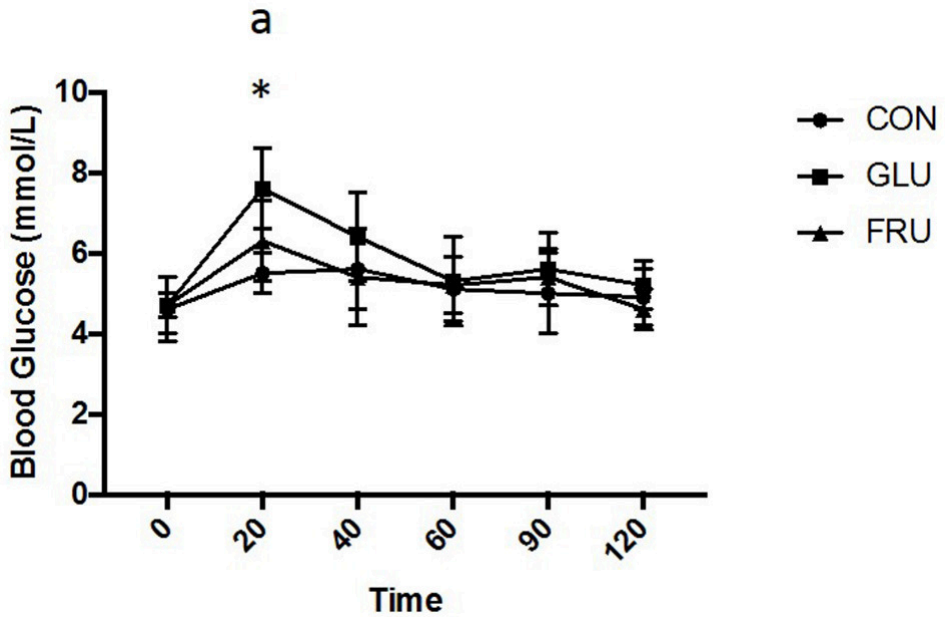
Blood glucose concentration (mmol/L) during CON, GLU and FRU trials. “*” indicates significantly different from baseline during GLU trial and “a” indicates significant difference, using *post-hoc* paired *t*-tests, between GLU and CON trial. Data are Mean ± SD.

Two factor repeated measures ANOVA demonstrated a main effect of trial (d.f. = 1.497; *F* = 7.931; *P* = 0.014), a main effect of time (d.f. = 2.030; *F* = 10.231; *P* = 0.002) and an interaction effect (d.f. = 3.191; *F* = 3.349; *P* = 0.038) for blood glucose (Figure 3). Subsequent analysis demonstrated that blood glucose was elevated from baseline values 20 min after ingestion on GLU and FRU. Blood glucose was higher 20 min after ingestion on GLU (*P* = 0.002) compared to CON and tended to be higher compared to FRU (*P* = 0.073). iAUC was 618 ± 74, 706 ± 70, and 642 ± 73 mmol/L 2 h (d.f. = 1.512; *F* = 6.469; *P* = 0.023) for CON, GLU, and FRU respectively and tended to be higher (*P* = 0.078) on GLU compared to CON.

**Figure 4:**
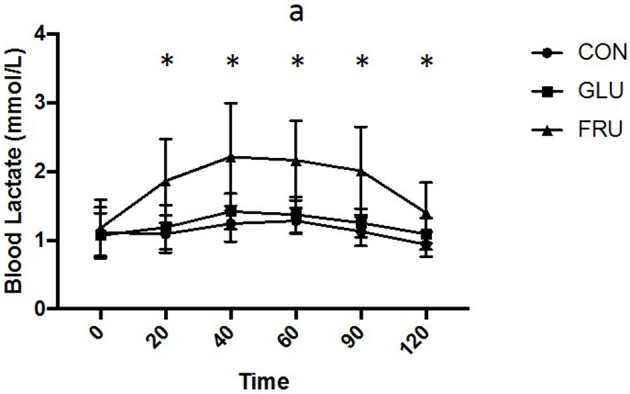
Blood lactate concentration (mmol/L) during CON, GLU and FRU trials. “*”indicates significant difference between FRU and CON trial and “a” indicates significant difference, using *post-hoc* paired *t*-tests, between FRU and GLU trial. Data are Mean ± SD.

Two factor repeated measures ANOVA demonstrated a main effect of trial (d.f. = 1.655; *F* = 12.146; *P* = 0.003), time (d.f. = 2.379; *F* = 14.007; *P* < 0.001) and interaction (d.f. = 3.659; *F* = 3.894; *P* = 0.017) for blood lactate (Figure 4). Blood lactate levels were higher (*P* < 0.05) on FRU compared to control at all time points after ingestion, were higher (*P* = 0.046) compared to GLU 60 min after ingestion and tended to be higher on GLU 40 min (*P* = 0.064) and 90 min (*P* = 0.064) after ingestion. iAUC was 138 ± 26, 151 ± 23, and 228 ± 63 mmol/L 2 h (d.f. = 1.507; *F* = 13.666; *P* = 0.003) and was greater on FRU compared to CON (*P* = 0.008) and GLU (*P* = 0.046).

Two factor repeated measures ANOVA demonstrated no main effect of trial (d.f. = 1.461; *F* = 0.131; *P* = 0.816), time (d.f. = 1.259; *F* = 2.976; *P* = 0.122), or interaction (d.f. = 2.464; *F* = 0.510; *P* = 0.647) for serum triglycerides. iAUC was 146 ± 68, 141 ± 85, and 137 ± 89 mmol/L 2 h (d.f. = 1.497; *F* = 0.091; *P* = 0.862).

## Discussion

The results of this study demonstrate that gastric emptying rate of fructose and glucose is similar when added to a semi-solid meal. In addition, fructose leads to an increase in blood lactate concentration and a small transient increase in blood glucose whereas the ingestion of glucose leads to an increase in blood glucose concentration only. No differences in appetite or triglyceride response were observed between the monosaccharides.

The potential role of dietary fructose in the development of a variety of chronic diseases has received much attention recently due to the rapid increase in consumption that has been observed in developed countries (1). The rate at which a monosaccharide is emptied from the stomach is of importance as this determines the rate at which it is available for absorption from the small intestine and the subsequent glycaemic response that occurs. Previous studies have demonstrated that increasing the glucose concentration of a liquid solution decreases the rate at which it empties from the stomach (5) and that, at high quantities of glucose ingestion, osmolality may also be an important consideration (21). Horowitz et al. (6), Yau et al. (7), and Yau et al. (8) have demonstrated that fructose empties from the stomach at a faster rate than an equivalent amount of glucose when ingested in a liquid form. It is important to study the effects of monosaccharide ingestion in liquids because fructose, sucrose, and high fructose corn syrup are often added to drinks as a sweetener. However, fructose is also added to solid and semi-solid food. The gastric emptying characteristics of solids are different to liquids (9) and ~35% of the variation in glycaemic response (20) can be atttributed to gastric emptying rate. Consequently, it is important to consider whether the food matrix in which a monosaccharide is ingested influences the gastrointestinal and metabolic response that follows. To the authors knowledge, this is the first study to have investigated the effect of glucose and fructose addition to a semi-solid meal. The results demonstrate that, when provided in this manner, the gastric emptying rate of these monosaccharides is similar and consequently the food is an important consideration when investigating the effects of nutrient ingestion on gastrointestinal function.

One of the weaknesses of this study is the relatively small number of participants that were recruited. However, a *post-hoc* power analysis with 80% power and an alpha level of 0.05 suggested that only six participants were needed to observe a main effect on half emptying time. The main question addressed in this study was whether the addition of glucose to the test meal resulted in differences to when fructose was added. Average half emptying time on the glucose trial was only 1 min different to the fructose trial indicating very little difference in this parameter. Indeed, *post-hoc* analysis suggests that over 1,000 participants may be required for this to reach statistical significance. The reason for the difference in previously reported observations in liquids compared to this study in semi-solids is an interesting avenue for future research.

In this study, the ^13^C sodium acetate breath test was used to assess the gastric emptying characteristics of the semi-solid meals. The benefit of this technique is that it provides a non-invasive measurement of gastrointestinal function. However, as a result, it provides an indirect measurement of the variable of interest. The ^13^C breath test for gastric emptying measurement of semi-solids has been shown to be a reliable indicator of gastric emptying rates when compared to scintigraphy in both adults (22) and children (23). T_lag_ and T_half_ times, calculated from ^13^C:^12^C ratios, are often higher than other methods as the technique is based on oxidation of the ingested substrate not just gastrointestinal processing. Braden et al. (22) observed that mean T_1/2_ time of an ingested semi-solid meal, consisting of 200 kcal, was 24 ± 11 min using scintigraphy but was calculated to be 78 ± 14 min using the ^13^C breath test. Hellmig et al. (24) determined expected gastric emptying rates of a fluid test meal and a solid test meal in 90 healthy volunteers using the ^13^C sodium acetate and ^13^C octanoic acid tests respectively. T_1/2_ and T_lag_ ranges were 43–151 and 10–73 min in response to the fluid challenge and were 51–429 and 29–203 min in response to the solid challenge. The results presented in this manuscript fall within these expected values and, as such, represent an accurate assessment of gastric emptying characteristics using this method of analysis.

A secondary purpose of this study was to examine the impact of addition of the monosaccharides on appetite response when ingested in semi-solid form. Gastric distension is known to effect satiety and satiation (3) and the addition of fructose to a liquid has been shown to reduce leptin concentrations (13, 14) and effect brain centers involved in food preferences (15). No differences between the trials was observed in subjective feelings of appetite, fullness or prospective food consumption, which is consistent with the gastric emptying observations presented. This provides further evidence that the effects of monosaccharide ingestion should consider the food matrix in which sugars are delivered. An important observation is that there was also no difference in appetite response between either of the monosachharide trials and the control trial. This could be due to the statistical power of this study. However, from Figures 1, 2, any statistical differences that may occur are likely to be small and of short duration, thus unlikely to be physiologically or clinically significant. It appears that the addition of 40 g of monosaccharide has little acute effect on acute appetite response when ingested in the manner described despite the increase in caloric load and there was no difference observed between individual monosaccharides.

Previous studies have demonstrated that the ingestion of fructose results in an increase in blood glucose, insulin and GLP-1 (11) but to a lesser extent than an isocaloric glucose trial as well as a greater increase in circulating triglycerides (12). These observations are from studies that have involved ingestion of large quantities [75 g in Kong et al. (11) and 0.75 g/kg body mass in Chong et al. (12)] of a single monosaccharide in liquid form. Other studies, such as (16), have shown that the ingestion of smaller quantities (36 g) of fructose have less of an effect on these metabolic markers. The results of the present study support the observation that the ingestion of fructose increases blood glucose concentration but to a lesser extent than when glucose was added to the semi-solid meal as both trials demonstrated significant elevations in blood glucose concentration 20 min after ingestion and this tended to be higher when glucose was ingested when compared to fructose. An important consideration is that, unlike those which involve liquid ingestion, the monosaccharide in this study was added to a meal that already contained significant quantities of carbohydrate. Consequently, all observations are the effect of total nutrient ingestion rather than solely the effect of the added glucose or fructose. Although not measured in this study, it is therefore likely that GLP-1 and insulin concentrations were also elevated in the glucose trial to a greater extent than when fructose was ingested.

The results of the present study demonstrate that serum lactate concentration was increased when fructose was ingested, however, this was not the case during the control or glucose trials. This observation has been reported when fructose is added to liquids (16) and tracer studies demonstrate that up to a quarter of dietary fructose may be converted to lactate (25). The reason for this appears to be so that carbon atoms derived from fructose can be released from the liver for utilization. An interesting observation was that post-fructose serum lactate concentrations demonstrated a large degree of variability with standard deviations ranging from 0.4 to 0.8 mmol/L whereas post-glucose ingestion this variability was much less (standard deviations of 0.2–0.3 mmol/L). This observation appears to be in agreement with other studies that involving fructose ingestion in a liquid form (8, 16). Similarly, other studies (26, 27) appear to show a degree of variability in plasma lactate response during exercise with ingestion of a combined glucose-fructose solution. The reason for this observation is unclear however is likely to be due to individual differences in ability to convert ingested fructose to lactate. This observation warrants further study and, in particular, whether differences in normal dietary intakes of fructose, or fructose-containing sugars, effects post-ingestion blood lactate concentration.

While many studies have demonstrated that ingestion of large quantities of fructose delivered via liquids within a short time period lead to a greater increase in triglycerides than similar quantities of glucose (12, 13), the results of the present study showed no difference in triglyceride response between trials with iAUC being nearly identical between all three trials. This observation warrants further investigation.

## Conclusion

In conclusion, the results of this study suggest that there is no difference in gastric emptying rate of a semi-solid meal containing additional glucose or fructose. This is in contrast to other literature in this area which has suggested that gastric emptying rate of fructose is faster than glucose when added to liquids This would suggest that the addition of sugars to solids, semi-solids and liquids is handled differently by the stomach and, therefore, delivery to the intestine for absorption may be affected. This indicates that the food matrix in which a monosaccharide is provided is an important consideration when studying the impact of simple sugars on metabolic response as the rate of delivery to the intestine is an important consideration for appearance in the circulation.

No difference in appetite response between sugars was observed suggesting that the ingestion of fructose, in quantities provided in this study, is unlikely to lead to greater food ingestion than glucose. Indeed, the addition of either sugar seemed to have little impact on appetite response compared to the control trial despite the increased caloric intake. This warrants further consideration in terms of focusing on the addition of individual monosaccharides to food products is or whether reducing total sugar should be the main focus.

Similar to other literature, the addition of fructose to a semi-solid meal leads to smaller increases in blood glucose, and presumably insulin, and larger increases in blood lactate than when glucose is added. An interesting avenue for future research in this area is elucidation of the reasons for the relatively high degree of variability in lactate response post-fructose ingestion. In contrast to some other literature, there appears to be no difference in triglyceride response between sugars ingested in this manner over a 2 h time period. An important consideration for future study here is the quantity of fructose provided as this was lower than other studies in this area but possibly more representative of normal dietary intakes. Further research in this area would assist in determining the potential role ingestion of fructose may have in a number of chronic disease states.

## Ethics statement

This study was carried out in accordance with the recommendations of The Faculty of Science and Engineering Ethical Advisory Committee at Manchester Metropolitan University with written informed consent from all subjects. All subjects gave written informed consent in accordance with the Declaration of Helsinki. The protocol was approved by the The Faculty of Science and Engineering Ethical Advisory Committee at Manchester Metropolitan University.

## Author contributions

GE, JM, and AY conceived and designed the experiment. GE and AY performed data collection. GE analyzed the data and wrote the paper with contributions from JM and AY. All authors have read and approved the final manuscript.

### Conflict of interest statement

The authors declare that the research was conducted in the absence of any commercial or financial relationships that could be construed as a potential conflict of interest.
